# Augmenting Transport versus Increasing Cold Storage to Improve Vaccine Supply Chains

**DOI:** 10.1371/journal.pone.0064303

**Published:** 2013-05-22

**Authors:** Leila A. Haidari, Diana L. Connor, Angela R. Wateska, Shawn T. Brown, Leslie E. Mueller, Bryan A. Norman, Michelle M. Schmitz, Proma Paul, Jayant Rajgopal, Joel S. Welling, Jim Leonard, Sheng-I Chen, Bruce Y. Lee

**Affiliations:** 1 Public Health Computational and Operational Research (PHICOR) Group, School of Medicine and Graduate School of Public Health, University of Pittsburgh, Pittsburgh, Pennsylvania, United States of America; 2 Pittsburgh Supercomputing Center, Pittsburgh, Pennsylvania, United States of America; 3 Department of Industrial Engineering, School of Engineering, University of Pittsburgh, Pittsburgh, Pennsylvania, United States of America; University of Florida, United States of America

## Abstract

**Background:**

When addressing the urgent task of improving vaccine supply chains, especially to accommodate the introduction of new vaccines, there is often a heavy emphasis on stationary storage. Currently, donations to vaccine supply chains occur largely in the form of storage equipment.

**Methods:**

This study utilized a HERMES-generated detailed, dynamic, discrete event simulation model of the Niger vaccine supply chain to compare the impacts on vaccine availability of adding stationary cold storage versus transport capacity at different levels and to determine whether adding stationary storage capacity alone would be enough to relieve potential bottlenecks when pneumococcal and rotavirus vaccines are introduced by 2015.

**Results:**

Relieving regional level storage bottlenecks increased vaccine availability (by 4%) more than relieving storage bottlenecks at the district (1% increase), central (no change), and clinic (no change) levels alone. Increasing transport frequency (or capacity) yielded far greater gains (e.g., 15% increase in vaccine availability when doubling transport frequency to the district level and 18% when tripling). In fact, relieving all stationary storage constraints could only increase vaccine availability by 11%, whereas doubling the transport frequency throughout the system led to a 26% increase and tripling the frequency led to a 30% increase. Increasing transport frequency also reduced the amount of stationary storage space needed in the supply chain. The supply chain required an additional 61,269L of storage to relieve constraints with the current transport frequency, 55,255L with transport frequency doubled, and 51,791L with transport frequency tripled.

**Conclusions:**

When evaluating vaccine supply chains, it is important to understand the interplay between stationary storage and transport. The HERMES-generated dynamic simulation model showed how augmenting transport can result in greater gains than only augmenting stationary storage and can reduce stationary storage needs.

## Introduction

When addressing the urgent task of improving vaccine supply chains, there is often a heavy emphasis on stationary storage [1]. To guide the assessment and improvement of vaccine supply chains, the World Health Organization (WHO) created the Effective Vaccine Management (EVM) tool and PATH collaborated with the WHO and the United Nations Children's Fund (UNICEF) to develop the Cold Chain Equipment Manager (CCEM) tool [2], [3]. While both tools are helpful in assessing a country's supply chain, both focus more on stationary storage rather than transport aspects of supply chains. International donors have also responded to the growing needs of restricted cold chains by donating equipment, often in the form of stationary storage devices [4]–[6]. The recent and impending introductions of new vaccines have prompted many countries to examine their vaccine supply chains (i.e., the system and series of steps required to get vaccines from the manufacturers to the people). For example, the introduction of pneumococcal vaccine caused the storage space requirement in the Turkey vaccine supply chain to quadruple [7].

However, transport is a major component of vaccine supply chains and can be a source of bottlenecks. Rotavirus vaccine introduction overwhelmed both storage and transport capacities of vaccine supply chains in several Latin American countries in 2006 and 2007 [8]. Many low- and middle-income countries face difficulties in maintaining efficient vaccine supply chains with the current routine immunization regimen, let alone with any of the 12 new, bulkier vaccines that are proposed for introduction by 2019 and are expected to create bottlenecks in both storage and transport aspects of vaccine supply chains [1]. The recent availability of new vaccines, such as pneumococcal and rotavirus, can relieve the burden of disease for millions of infants worldwide, but if vaccine cold supply chains cannot accommodate the increased volume required to ensure adequate supply, populations will not receive these benefits. As new vaccines are introduced, transport constraints will become an increasing issue in vaccine supply chains.

It remains unclear whether adding cold storage or augmenting transport elicits a greater improvement in vaccine availability. It is imperative to evaluate vaccine supply chains as soon as possible to prevent disruptions, as many vaccine supply chains are already restricted by limited capacity in either transport or storage at certain levels [1]. Determining the effects of augmenting transport can be challenging without using a dynamic analysis, such as a simulation model. Therefore, this study utilized a dynamic computational model of Niger's vaccine supply chain to compare the effects of introducing cold storage and altering transport frequency at various levels. The objective was to determine whether adding cold storage devices or increasing transport frequency would have a greater impact and to identify the locations where these additions would be most beneficial, as measured by vaccine availability.

## Methods

This study utilized HERMES, the Highly Extensible Resource for Modeling Supply Chains, a software package developed by the HERMES Modeling Team, to generate a discrete event simulation model of the Niger vaccine supply chain. The HERMES-generated model represents all logistical components of a supply chain, including the number and size of transport and storage devices, shipment policies, delivery and order frequencies, packaged vaccine size within the supply chain and vaccine storage temperatures, the number of vaccines traveling through the system, and the routes of the transport vehicles. Previous publications have described this model in detail [9]–[11].

### Niger Vaccine Supply Chain

**Figure 1:**
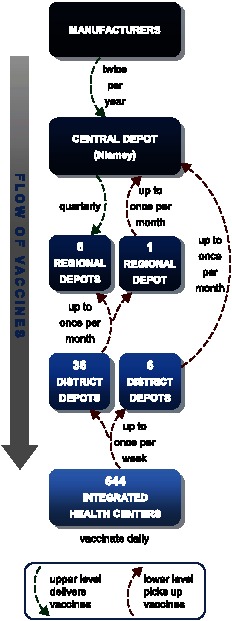
Niger vaccine supply chain network.

Figure 1 depicts the structure of the entire Niger vaccine supply chain. Data to construct this network came from direct field observations in Niger and personal communications with members of the following organizations: the WHO in both Geneva and the Niger country office in Niamey, the Niger Ministry of Health (MOH), the UNICEF Niger country office, the Niger National Geographic Institute (NGI), and the Expanded Program on Immunization (EPI) in Niger. The vaccine supply chain consists of four levels, whose functional units include one central depot in the capital city of Niamey, seven regional depots, 42 district depots, and 644 integrated health centers (IHCs) throughout the country. Vaccine administration occurs daily and only at the IHC level. The number of IHCs per district ranges from 5 to 36. Most of the IHCs are in the south of Niger, where the majority of the population resides.

### Transport

The central depot receives vaccines from the manufacturers *via* UNICEF twice per year. The regional level receives vaccines from the central depot. All but one regional depot receive shipments by cold trucks on a fixed, quarterly schedule. The remaining regional depot, in Niamey, retrieves vaccines by pick-up truck as often as once per month, as needed, due to its close proximity to the central depot. The district level picks up vaccines from the regional level, except for six district locations which pick up vaccines directly from the nearby central depot. The districts retrieve vaccines by pick-up truck as often as once per month, as needed. IHCs equipped with cold storage capacity retrieve vaccines monthly using up to two 2.5L vaccine carriers but can pick up vaccines more often – up to once a week, as needed. Districts and IHCs order enough vaccines to meet average monthly demand, including an allowance for open vial waste as well as an additional 25% buffer.

### Cold Storage

Based on a previously performed inventory, each vaccine storage location has a number of refrigerators (2°C to 8°C) and freezers (−15°C to −25°C) dedicated to EPI operations, with pre-defined storage capacities based on the make and model of the registered unit. Walk-in refrigerators and freezers are utilized at the central depot and three of the regional level depots while the other regional, district, and IHC locations operate with conventional upright or supine refrigerators and freezers. Table 1 summarizes the devices that store and transport EPI vaccines in Niger.

**Table 1:** Characteristics of cold storage and transport equipment by supply chain level.

	Stationary storage equipment		Transport equipment	
Supply chain level	Stationary storage device (quantity)	Average net volume per device, liters (range)	Transport device (quantity)	Net volume per device, liters
Central	Cold room (2)	18,000L (16,000–20,000L)	Cold truck (2)	9293L
Regional	Cold room (3)	13,333L (12,000–16,000L)	Pick-up truck (1)	4 large cold boxes, 172L
	Refrigerator (20)	120L (11–378L)	Large cold box (4)	43L
District	Refrigerator (119)	76L (11–378L)	Pick-up truck (42)	8 cold boxes, 176L
			Cold box (336)	22L
Integrated health center	Refrigerator (723)	35L (11–169L)	Vaccine carrier (1284)	2.5L

### HERMES-generated Simulation Model of Niger Supply Chain

Net capacity of each cold device was determined by its size and utilization (i.e., the percentage of gross physical space that can actually be used after accounting for space occupied by shelving and the inability to pack vaccines with no space between items). The data gathered from in-country personnel helped construct the vaccine shipping policies with realistic delivery occurrences between locations. The simulated shipments do not contain more vaccines than the cold transport device can hold. A location requesting vaccines in excess of the amount contained in one shipment must wait for the next shipment to fulfill the vaccine delivery request.

### Vaccine Characteristics

The model represents each vaccine vial with a computational entity and the flow of all WHO-EPI vaccines flowing through the supply chain simultaneously, including the six current EPI vaccines [Bacille Calmette-Guérin (BCG), diphtheria-tetanus-pertussis-hepatitis B-Haemophilus influenza type B (DTP-HepB-Hib), oral polio (OPV), measles (M), tetanus toxoid (TT), and yellow fever (YF)] and the new Prevnar 13 pneumococcal (PCV) and Rotarix rotavirus (RV) vaccines, which have been approved for funding to become regular EPI vaccines in Niger in 2013 and 2014, respectively [12]. Under the regimen of one BCG dose at 1.9 cm^3^ (packed volume, including diluent), three DTP-HepB-Hib doses each at 16.8 cm^3^, four OPV doses each at 1.0 cm^3^, one M dose at 2.6 cm^3^, two TT doses each at 3.0 cm^3^, and one YF dose at 8.5 cm^3^, the current EPI schedule requires 73.4 cm^3^ to fully immunize one child [13]–[15]. With the addition of three PCV doses each at 12 cm^3^ and two RV doses each at 17.1 cm^3^ to the EPI, this volume will increase to 143.6 cm^3^ [14].

### Population Demand

The model uses a projected population demand for 2015, based on district-level census data collected in 2005 which was inflated to conform to estimated pregnant woman, newborn, and surviving infant cohorts in the Comprehensive Multiyear Plan (cMYP) for Niger [16]. Each district population was evenly distributed among the IHCs to determine the number of individuals in the specified demographic groups arriving at IHCs for vaccines. Target vaccination coverage rates were used as outlined in the cMYP: 95% for BCG, DTP-HepB-Hib, OPV, M, TT, YF, and PCV; and 70% for RV [16]. The resulting median number of doses required to meet these coverage rates at an IHC location in a given month was 2088 with a maximum of 4358.

### Supply Chain Performance Measure

The overall objective of this investigation was to maximize the number of vaccines available for the demand across all locations, time periods, and vaccine types. Vaccine availability, the primary output measure, expresses the percentage of patients that could be vaccinated based on the number of vaccines available at the IHCs. The vaccine availability is calculated as follows:
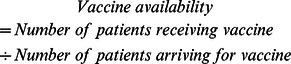
Missed vaccination opportunities occur when individuals arrive for vaccines but the appropriate vaccines are not available, resulting in vaccine availability of less than 100%.

### Simulation Experiments

Each simulation represented one year of vaccine supply chain operations. The first set of experiments added additional storage capacity to locations in the Niger vaccine supply chain. This entailed identifying the maximum amount of storage space required at each location based on current ordering and shipping policies. A location experiences storage constraints if the maximum storage volume it might require is greater than the volume it has at baseline. The experiments compared the effects on vaccine availability of relieving storage constraints in different supply chain levels while maintaining current transport policies. The experiments also alleviated all storage constraints at every location in the supply chain. The resulting vaccine availabilities indicated at which locations alleviating storage constraints would provide the greatest benefit to the supply chain as well as the maximum effect that added storage can achieve.

The second set of experiments used existing storage capacities and increased in-country transport frequencies between various levels. Doubling the number of possible trips between the central and regional levels required altering the fixed schedule to deliver vaccines to the regional depots eight, rather than four, times per year and allowed the Niamey regional depot to retrieve vaccines from the central depot up to twice per month. Doubling the number of possible trips delivering to the district and IHC levels allowed the district depots to retrieve vaccines up to twice per month and allowed the IHCs to retrieve vaccines twice per month and as often as twice per week. The experiments initially doubled the frequency of vaccine delivery to one level at a time. The experiments also studied the effects of doubling the frequency between all levels of the supply chain at once.

The subsequent transport experiments used tripled transport frequencies, such that the Niamey regional depot could retrieve vaccines up to three times per month and all other regional depots would receive vaccines once per month. District depots could retrieve vaccines up to three times per month, and IHCs could retrieve vaccines up to three times per week. These experiments provided vaccine availabilities that identified where along the supply chain additional transport would most improve the flow of vaccines to the IHCs and allowed a comparison of the benefits of doubling *versus* tripling transport frequency. This study also compared the magnitude of improvement in vaccine availability between added storage and added transport.

Locations receiving vaccines at an increased frequency required fewer vaccines to supply them until the following shipment, so the sizes of affected shipments reduced accordingly. The experiments identified and alleviated storage constraints under doubled and tripled transport frequencies to measure the degree to which increasing transport frequency affects the amount of stationary storage required. The vaccine availability at IHCs and the additional storage volume used across all locations in the supply chain were compared under baseline, doubled, and tripled transport frequencies.

## Results

### Adding Stationary Storage Capacity Alone

**Figure 2:**
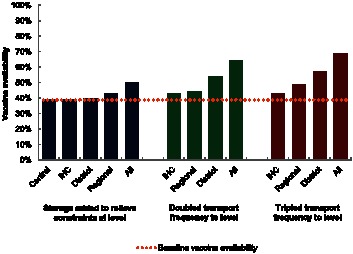
Vaccine availability after addition of storage or transport capacity.

Figure 2 displays the resulting vaccine availabilities after storage or transport was added at each level. At baseline, for a 2015 Niger population with PCV and RV introductions, the vaccine availability across all IHCs was 39%. The central depot was highly constrained, needing more than 99% of its available 36,000L of net refrigerated storage capacity. An additional 52,948L of net storage relieved storage constraints at this location. Three of seven regional depots did not have a cold room at baseline and experienced storage constraints, each of which required an additional 1,155L to 4,186L. At the district level, 19 of the 42 depots required an additional 3L to 319L to relieve storage constraints. Only eight of the functional IHCs experienced storage constraints at baseline, requiring an additional 1L to 5L. Relieving storage constraints at upper levels allowed more vaccines to flow to lower levels, creating additional bottlenecks. Therefore, relieving storage constraints throughout the supply chain required more added capacity than the sum of the capacities needed at each individual level.

Relieving storage constraints only at the regional level increased vaccine availability by 4%. Adding storage to relieve only constrained district depots increased vaccine availability by 1%. Bottlenecks in storage and transport at other levels of the supply chain prevented the addition of any amount of storage to the central or IHC levels alone from having any effect on vaccine availability. Adding enough cold storage capacity to relieve storage constraints for the entire supply chain increased vaccine availability by 11%.

### Increasing Transport Frequency Alone

Doubling the frequency of scheduled trips delivering vaccines from the central depot to the regional depots from four times to eight times per year, while also doubling the frequency at which the Niamey regional depot is able to retrieve vaccines from the central depot from once to twice per month, increased vaccine availability by 5%. Doubling the frequency at which districts were able to retrieve vaccines from once per month to twice per month increased vaccine availability by 15%. Allowing IHCs to retrieve vaccines from districts up to twice per week, rather than once per week, increased vaccine availability by 4%. Doubling the shipping frequency across the entire supply chain increased vaccine availability by 26%.

Tripling the frequency of trips delivering from the central depot to the regional level increased vaccine availability by 10% as compared to baseline. Tripling the frequency at which districts were able to retrieve vaccines increased vaccine availability by 18%, only a slightly higher increase than was achieved by doubling the frequency. Allowing IHCs to retrieve vaccines from districts up to three times per week increased vaccine availability by 4%, the same benefit achieved under doubled shipping frequency to the IHC level. Tripling the shipping frequency across the entire supply chain increased vaccine availability by 30%.

While adding cold storage capacity to the regional level can increase vaccine availability by up to 4%, doubling the possible number of trips delivering to the district level can increase vaccine availability by up to 15%. If no equipment is added to the current vaccine supply chain by 2015, the vaccine supply chain will have the ability to supply only 39% of the needed vaccinations (current EPI with PCV and RV introduced). Based on cMYP [16] population projections, increasing vaccine availability by 1% means that >135,000 more vaccinations could be provided in 2015. A 5% increase translates to >677,000 more vaccinations.

### How Increasing Transport Affects Storage Capacity Requirements

**Figure 3:**
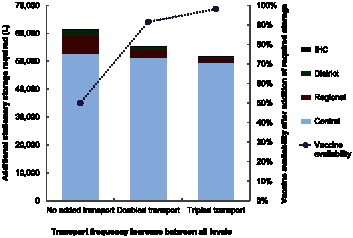
Additional stationary storage requirement under increased transport frequencies.

As Figure 3 shows, increasing transport frequency not only resulted in higher vaccine availability but also reduced the amount of stationary storage required. With no added transport, the entire supply chain required an additional 61,269L of storage to relieve constraints. Doubling transport frequency reduced this storage need to 55,255L. Tripling transport frequency further reduced it to 51,791L. Relieving storage constraints while simultaneously doubling transport frequency increased vaccine availability by 42%. Tripling transport frequency while relieving storage constraints increased vaccine availability by 48%, thus providing 98% of the vaccinations requested at IHCs.

## Discussion

Although relieving storage constraints may be important, increasing shipping frequency to the district level alone resulted in a far greater improvement in vaccine availability than the addition of any amount of storage to all levels of the supply chain. Adding storage capacity at the central or IHC levels without adding transportation had little to no impact on Niger's overall vaccine availability. Both storage and transport capacity constraints, particularly at the regional and district levels of the vaccine supply chain, created significant bottlenecks that prevented the effective flow of vaccines to the IHC level. Identifying these areas of restriction allows for the most effective distribution of required new cold capacity. The most effective addition of storage capacity occurred at the regional level. Though the central level requires a far greater amount of cold volume in order to become unconstrained, adding a fraction of that space to the regional level will bring more relief to the currently overburdened supply chain. These results suggest that optimal space allocation is not easily determined by the area with the greatest deficit but is instead strongly influenced by dynamic downstream interactions.

Overlooking transport may be ignoring a vital component of vaccine supply chains. In Niger, there appears to be a ceiling effect for vaccine availability when only adding cold storage. In fact, augmenting transport would substantially decrease storage requirements and may not require further capital investment. If vehicles are already available, then this may instead involve additional fuel and labor or different vehicle and labor allocation strategies. If more trips for each vehicle are not feasible, then replacing existing vehicles with larger ones (e.g. substituting trucks for motorbikes) or adding more of the same vehicles could be considered, although this may depend on how accessible locations may be by large vehicles. Alternatively, implementing (or redesigning) delivery loops, where each truck would deliver vaccines to multiple locations in a single trip could be feasible.

Understanding the role and impact of transport can be difficult without dynamic simulation modeling [17]–[19]. While static models can analyze storage aspects of a supply chain, they may struggle to represent the complex interplay between storage and transport. Relieving some storage or transport constraints can exacerbate or alleviate bottlenecks at other locations in the supply chain. When adding capacity to highly constrained locations, particularly those at the upper levels of the supply chain, one must consider the consequences for facilities that receive vaccines from these locations. Adding storage capacity can, in some cases, alleviate transport constraints, as less frequent trips are necessary when a location can store a longer-term supply of vaccines. Conversely, increasing transport frequency between two levels can alleviate storage constraints at both levels, as fewer vaccines must be stored at these locations at any one time. When planning to increase the cold capacity of a vaccine supply chain, allocating new resources to have the greatest impact seems justified. Dynamic modeling can provide insight into where these resources can save the most lives.

Some organizations have emphasized the importance of transport; Transaid, Riders for Health, and VillageReach improve and expand transport systems to increase access to healthcare and other essential services in low- and middle-income countries. Transaid develops vehicle management and maintenance systems in areas such as vaccine cold chain transport logistics [20]. Riders for Health trains drivers and provides preventative maintenance on vehicles commissioned by health organizations [21]. VillageReach addresses distribution systems for health resources, including vaccine cold chain transport infrastructure [22]. These organizations address the many aspects of transport that affect vaccine availability, including vehicle availability and road safety. Efforts such as these are essential for new and existing vaccines to reach the people they are intended to protect.

## Limitations

All computer models make simplifying assumptions and cannot represent all possible factors or outcomes [23]–[25]. For this analysis, while model assumptions and data inputs were drawn from extensive review of the literature and data collection, the sources may vary in quality and model parameters may not hold under all conditions. The findings of this study suggest that when seeking to improve the vaccine supply chain for any country, transport should be a consideration. While this study examined the effects of augmenting only in-country transport, increasing the frequency of shipments from vaccine manufacturers to the central depot would also likely have positive implications for vaccine availability. However, not all countries are the same in their supply chain needs and population demand. Some may benefit more or less from changes in transport. It is possible that a given country may only have storage constraints. Future research can study constraints and alleviation strategies for vaccine supply chains in other countries.

## Conclusions

Cold capacity in country vaccine supply chains may need to expand to meet increasing demands due to growing populations, new vaccine introductions, and larger packaging. While there has been an emphasis on donating stationary cold storage, augmenting transport may be just as crucial. In fact, as this study has found in Niger, increasing transport may have a far greater impact on vaccine availability than adding only stationary storage capacity. Furthermore, increasing transport could substantially decrease stationary storage requirements. Dynamic simulation modeling of vaccine supply chains can elucidate the complex interplay between storage and transport and guide donor priorities.
