# Correction: Augmenting Transport versus Increasing Cold Storage to Improve Vaccine Supply Chains

**DOI:** 10.1371/annotation/b428dad2-e829-4289-acae-c44369d55a80

**Published:** 2013-12-10

**Authors:** Leila A. Haidari, Diana L. Connor, Angela R. Wateska, Shawn T. Brown, Leslie E. Mueller, Bryan A. Norman, Michelle M. Schmitz, Proma Paul, Jayant Rajgopal, Joel S. Welling, Jim Leonard, Sheng-I Chen, Bruce Y. Lee

Figures 1, 2, and 3 have been updated for better readability. Please see the correct figures here:

Figure 1: http://plosone.org/corrections/pone.0064303.g001.cn.tif

Figure 2: http://plosone.org/corrections/pone.0064303.g002.cn.tif

Figure 3: http://plosone.org/corrections/pone.0064303.g003.cn.tif

The affiliation for author Bruce Lee has been updated. The updated affiliation is: Public Health Computational and Operational Research (PHICOR) Group, Johns Hopkins Bloomberg School of Public Health, Baltimore, Maryland, United States of America

The affiliation for Leila A. Haidari, Diana L. Connor, Leslie E. Mueller, and Michelle M. Schmitz has been updated. The correct affiliation is:

Public Health Computational and Operational Research (PHICOR) Group, Johns Hopkins Bloomberg School of Public Health, Baltimore, Maryland, United States of America 

